# Handling heatwaves: balancing thermoregulation, foraging and bumblebee colony success

**DOI:** 10.1093/conphys/coae006

**Published:** 2024-02-08

**Authors:** Tiffany Bretzlaff, Jeremy T Kerr, Charles-A Darveau

**Affiliations:** Department of Biology, University of Ottawa, 30 Marie Curie, Ottawa, ON, Canada K1N 6N5; Department of Biology, University of Ottawa, 30 Marie Curie, Ottawa, ON, Canada K1N 6N5; Department of Biology, University of Ottawa, 30 Marie Curie, Ottawa, ON, Canada K1N 6N5; Department of Biology, University of Ottawa, 30 Marie Curie, Ottawa, ON, Canada K1N 6N5

**Keywords:** Bombus, bumblebee colonies, castes, chronic heat stress, foraging, Heat tolerance, thermoregulation

## Abstract

Climate changes pose risks for bumblebee populations, which have declined relative to the growing frequency and severity of warmer temperature extremes. Bumblebees might mitigate the effects of such extreme weather through colonial behaviours. In particular, fanning behaviour to dissipate heat is an important mechanism that could reduce exposure of thermally sensitive offspring to detrimental nest temperatures (T_n_). The allocation of workers towards fanning over prolonged periods could impact foraging activity that is essential for colony-sustaining resource gathering. Colony maintenance and growth could suffer as a result of nutritional and high ambient temperature (T_a_) thermal stress. It remains uncertain whether a compromise occurs between thermoregulation and foraging under chronic, sublethal heat events and how colony success is impacted as a result. This study held colonies of *Bombus impatiens* at constant high T_a_ (25°C, 30°C or 35°C) for 2 weeks while quantifying the percentage of foragers, fanning incidence, nest temperature (T_n_) and other metrics of colony success such as the percentage of adult emergence and offspring production. We found that foraging and adult emergence were not significantly affected by T_a_, but that thermoregulation was unsuccessful at maintaining T_n_ despite increased fanning at 35°C. Furthermore, 35°C resulted in workers abandoning the colony and fewer offspring being produced. Our findings imply that heatwave events that exceed 30°C can negatively impact colony success through failed thermoregulation and reduced workforce production.

## Teaser

Climate change and heatwave events place urgency on understanding bumblebee colony responses to heat stress. We investigated the effect of chronic high ambient temperature exposure on colony success, where nest thermoregulation was unsuccessful, worker abandonment increased and fewer offspring were produced, providing insight into potential causes of observed bumblebee population declines.

## Introduction

Impacts of climate change, including current and projected increases in temperature (The Core Writing Team IPCC, 2015), are placing stress on ectotherms globally, exposing some to temperatures, which equal or exceed physiological thermal limits (Sunday *et al.,* 2014). Species inhabiting mid-latitudes, where temperature variation is greater, are likely to face greater heat stress challenges (Kingsolver *et al.,* 2013). More frequent and intense extreme heat events are predicted in the future (Meehl and Tebaldi, 2004), leaving ectotherms, whose body temperatures respond more directly to that of the environment, especially vulnerable to increases in ambient temperature (T_a_). One group of particular concern is insect pollinators, including bumblebees, who play important environmental and agricultural roles (see Klein *et al.,* 2006; Gill *et al.,* 2016). Various stressors are known to impact bumblebees, including pesticides (Mommaerts *et al.,* 2010; Whitehorn *et al.,* 2012; Bryden *et al.,* 2013; Alkassab and Kirchner, 2017) and parasites (Gegear *et al.,* 2005; Rutrecht and Brown, 2008) impacting individuals and colonies, as well as decreases in species richness linked to land use change (Vray *et al.,* 2019). Yet, climate change also contributes to the decline of bumblebee populations (Fourcade *et al.,* 2019; Soroye *et al.,* 2020) and to observed range losses (Kerr *et al.,* 2015). How the growing intensity and severity of extreme weather, such as heat waves, can alter bumblebee colony persistence is important to understanding these species' ability to cope with future alterations in global temperature.

Bumblebees are not typical ectothermic insects as they are capable of generating heat endogenously, allowing them to warm body temperatures for flight (Heinrich, 1972a, 1974, 1995; Staples *et al.,* 2004; Masson *et al.,* 2017) and adults possess high critical thermal limits which reflect this heat generation (i.e. Heinrich, 1976; Oyen and Dillon, 2018; Bretzlaff *et al.,* 2023). Moreover, bumblebees are social and workers collectively help maintain colonies through resource gathering and nest-wide thermoregulation. Thermoregulatory abilities of the individual warm up the colony when T_a_ falls, and behavioural mechanisms, such as fanning, help cool the nest when conditions are hotter. Fanning has been previously described in both bumblebees (Weidenmüller, 2004) and honeybees (Cook *et al.,* 2016) as a bee who remains in one location for period of 10 seconds while steadily fanning with spread wings. This mechanism aids in convective and evaporative heat loss within the colony (Vogt, 1986; Heinrich, 2004) where offspring, in particular, are vulnerable to heat. Worker bees must buffer these individuals from changes in T_a_ by maintaining relatively stable nest temperatures (T_n_) which ranges between 30°C and 33°C in bumblebees (Barrow and Pickard, 1985; Vogt, 1986; Schultze-Motel, 1991; Heinrich, 2004). Thermal stability of the nest appears to be species-specific, however, where long-term monitoring of colonies successfully established in aboveground artificial nests in the field show a range of brood temperature across species exposed to the same environmental temperature fluctuation (Gradišek *et al.,* 2023). Nonetheless, deviation from optimal temperatures alter development of communication, olfactory senses and short-term memory in honeybees (Tautz *et al.,* 2003; Groh *et al.,* 2004; Jones *et al.,* 2005; Wang *et al.,* 2016), as well as hinder emergence and reduce individual longevity in bumblebees (Groh *et al.,* 2004; Medrzycki *et al.,* 2010). Furthermore, chronic exposure to elevated temperature has been demonstrated to reduce worker size (Guiraud *et al.,* 2021), increase wing size variation of males (Gerard *et al.,* 2018) and hinder worker responses to stimuli (Perl *et al.,* 2022). Collective thermoregulation at both low and high T_a_ incurs significant energetic costs for individuals and the colony, yet under acute heat stress, increased fanning efforts do not result in successful nest thermoregulation (Vogt, 1986; Bretzlaff *et al.,* 2023). Thus, high upper critical temperature limits observed among adult bumblebees may not reflect the colony's susceptibility to negative effects of high temperatures. Whole-colony responses to sublethal chronic thermal stress may be critical for understanding their susceptibility to negative effects of rapidly warming climates.

The energetic costs and the ability to succeed in nest thermoregulation are not the only challenges encountered as a result of temperature variation. Bumblebees, unlike honeybees, lack age-caste division of labor, which partitions tasks among workers in different age classes. Instead, the tasks which help sustain a colony throughout its lifecycle, such as defense, foraging and other nest maintenance duties, are shared amongst all bumblebee workers and can be related to the size of the individual (Garófalo, 1978; Spaethe and Weidenmüller, 2002; Jandt and Dornhaus, 2009). These tasks may be prioritized based on current colony requirements (Free, 1955). For instance, about a third of a colony's workers participate in foraging (Brian, 1952; Stewart *et al.,* 2021) and previous experiments demonstrate that colonies may divert workers from foraging to nest incubation if provided with supplementary resources (Stewart *et al.,* 2021). However, observations under heat stress raise concerns surrounding allocating efforts between thermoregulation and the maintenance of a colony since acute T_a_ that exceeds optimal T_n_ conditions induces higher incidences of fanning while simultaneously reducing the incidence of nest maintenance (Vogt, 1986).

Colonies may be able to rebound from acute heat stress, but prolonged heat stress may pose a greater challenge. Chronic heat experiments have previously demonstrated reduced foraging (Kwon and Saeed, 2003) but other stressors, such as the quality of pollen diet, may interact to create more pronounced negative effects. For example, a combination of heat stress and poorly suitable diets causes decreased resource procurement and colony development, with greater variation in the percent mortality for bumblebee colonies of small size with about 60 workers (Vanderplanck *et al.,* 2019). These effects may have consequences for the long-term viability and success of a colony given that bumblebee density declines have been observed following heat waves (Rasmont and Iserbyt, 2012) with high air temperatures linked to reduced foraging trips (Couvillon *et al.,* 2010). Therefore, there is a need to understand not only the acute effects of high temperature stress on bumblebee colonies, but also the direct and indirect negative costs sustained if extreme heat events persist over longer periods.

The main objective of the present study was to test for direct and indirect effects of chronic high ambient temperature on colonies of a temperate North American species of bumblebee, *Bombus impatiens*. We subjected colonies to high T_a_ for 2-week periods and allowed them to forage within a temperature-controlled environment. We investigated whether chronic thermal stress induces workers to redirect their efforts from foraging to thermoregulation behaviours by monitoring the proportion of colony workers who foraged and the incidence of fanning behaviour. Furthermore, we simultaneously measured internal colony temperature to determine whether thermoregulatory efforts were successful and assessed colony success by quantifying adult emergence, mortality and offspring production, predicting that thermoregulation would be unsuccessful at high T_a_ and that the measures of colony success would be negatively affected. Our laboratory study will establish the capacity of endothermic colonial insects to cope with chronic heat stress.

## Materials and Methods

### Bumblebee colonies and holding conditions

Colonies of *Bombus impatiens* (Biobest Canada Ltd, Leamington, ON, Canada) were received 21 days prior to experimentation. On the day a colony arrived, the queen along with 10 workers were extracted and placed into a separate nesting box from the supplier along with approximately 3 brood clumps containing larvae and pupae. The new colony was given access to BIOGLUC® sugar solution from the supplier *ad libitum* and was immediately provided with two to three pollen balls to facilitate nest building. Thereafter, pollen was provided twice weekly where the colony was able to build new structures and increase population size until experimentation commenced.

### Experimental preparation

In order to monitor foraging behaviour of a colony under chronic thermal conditions, radio frequency identification (RFID) tags compatible with Microsensys Technology (Microsensys GmbH, Erfurt, Germany) were fixed to worker bees as follows: Subsequent to the 21-day colony recovery period, each bee was removed from the colony and incapacitated through refrigeration. Of the individuals collected, 43 were selected at random and each was fitted with an RFID tag on the dorsal side of the thorax, anterior to the wings using a small application of cyanoacrylate glue. This position did not restrict the movement nor the function of the wings and bees were able to engage in flight. The queen was returned to the experimental colony (without a tag) along with the 43 tagged workers.

Chronic, whole-colony heat exposure experiments required a temperature-controlled environment as well as ample space to allow for foraging. Experimentation thus took place in an environmental chamber on a 12:12 h light/dark cycle where temperature could be held constant. The relative humidity was not controlled but monitored and ranged from 38% to 44% across the range of temperature used, which is similar to the animal holding facility. An open container with approximately 5 L of water was placed in the chamber for the duration of the experiment; leftover water remained at the end of each trial. Many bumblebee species are known to inhabit aboveground nests, including artificial or human-made structures (Johnson *et al.,* 2019; Liczner and Colla, 2019), and similar artificial aboveground bumblebee nests have been shown to enable many species to successfully establish colonies in field settings (Gradišek *et al.,* 2023). No insulation was present inside the nest as it would prevent fanning observations, and although the presence of insulation within the nest reduces the cost of thermoregulation, nest temperature does not differ in similar settings (Bretzlaff *et al.,* 2023). A single experimental colony was placed in a 38 L Styrofoam cooler with a thickness of 1.5 cm to buffer direct exposure to the environmental chamber and similar to an artificial aboveground nesting scenario. A 60 × 60 × 180 cm flight cage, acting as a foraging destination for BIOGLUC® sugar solution, was connected to the colony via clear tubing with an internal diameter of 1.3 cm. This pathway between colony and flight cage spanned approximately 60 cm and also included two Microsensys RFID readers enabling outgoing and incoming bee movements to be recorded. Data collected by the readers were sent to a Microsensys iID controller where the digital information was stored. Prior to the collection of foraging data, colonies were allotted 3 to 5 days to discover the location of the sugar solution within the flight cage. Afterwards, the experiment was initiated by setting T_a_ within the environmental chamber to 25°C (25.00 ± 0.01, SE), 30°C (29.75 ± 0.05, SE) or 35°C (34.48 ± 0.07, SE) for a duration of 2 weeks; the temperatures used were based on a prior study assessing the cost of thermoregulation of the same species (Bretzlaff *et al.,* 2023). Pollen was supplied inside the nest box every second day of the experiment and the above procedure was repeated on 15 total colonies sorted into three different temperature groups (n = 5 per temperature).

### Quantification of foraging and fanning effort

The number of foraging trips gathered from RFID tag recordings over the 2-week period included date and time with direction being inferred from the Microsensys reader encountered first. From this data, a ‘foraging trip’ was chosen to be between 3.5 and 25 minutes based on initial observations during the pre-experimental setup and the size of the flight cage (e.g. some enter and exit the flight cage a few times but do not engage in foraging, while others collect sugar solution as well as search for additional sources before returning to the colony). A bee with 10 or more logged foraging trips was considered to be a ‘forager’ and the total number of trips taken by individuals who fall within this classification were gathered for analysis.

The incidence of individuals displaying fanning behaviour was estimated daily by capturing a 30-minute video of the top view of the colony covered by an acrylic transparent cover, an area corresponding to approximately 165cm^2^. To estimate the number of individuals fanning, the 1st, 5th, 10th, 15th, 20th, 25th and 30th minute of each video was reviewed, and the number of individuals fanning were counted. A ‘fanner’ was considered as a bee who remained stationary and beat their wings continuously for 10 seconds in a distinct posture with abdomen raised as described in (Weidenmüller, 2004; Cook *et al.,* 2016). The daily average number of fanners was calculated by summing the total number of fanners observed each day from a 30-minute video and using these sums to find the average across the 15 experimental days.

### Quantification of internal colony temperature

Internal colony temperature was quantified using two thermochron iButton® digital temperature loggers (iButtonLink Technology, Whitewater, WI, USA) placed among the brood cells with developing larvae inside the colony. The iButtons recorded temperature at 5-minute intervals for the duration of the 2-week experiment. Temperature data were extracted and averaged across the two iButtons both on a whole-trial as well as on a daily basis. Additionally, the temperature in the periphery of the nest away from the brood was monitored during initial trials.

### Quantification of adult emergence, mortality, abandonment and offspring production

Changes within a colony throughout an experimental trial were assessed by measuring various indicators. Adult emergence was defined as the percentage of adults which emerged from brood cells (untagged bees) during a 2-week exposure period. Mortality was quantified by the percentage of total bees who died before the end of an experimental trial. Abandonment of the colony was observed by some individuals who permanently moved to the flight cage. Therefore, this measure was included in analysis by quantifying the percentage of total bees who either were found deceased within the flight cage during the experiment or, were collected within the flight cage at the end. Finally, offspring production was assessed by counting larvae and pupae remaining within brood clumps at the end of each trial.

### Data analysis

Statistical analyses were performed in R (R Core Team, 2014) using experimental temperature as a categorical predictor in separate one-way ANOVAs with the following dependent variables: percent foragers, daily average number of fanners, average internal colony temperature, percentage of adult emergence, percent mortality, percent abandoned and total offspring. To determine whether the internal colony temperature or the number of fanners differed across the 14-day exposure period, linear mixed models were used with experimental temperature and day used as fixed effects and the tested colony as random effect. Pairwise analyses were conducted using the Tukey method and values are reported as mean ± standard error of the mean. For all variables, power analyses were conducted in Systat 13 using the mean values and average standard deviations obtained for each variable to determine sample sizes required for a power above 0.8.

## Results

### Effect of ambient temperature on foraging and fanning effort

**Figure 1:**
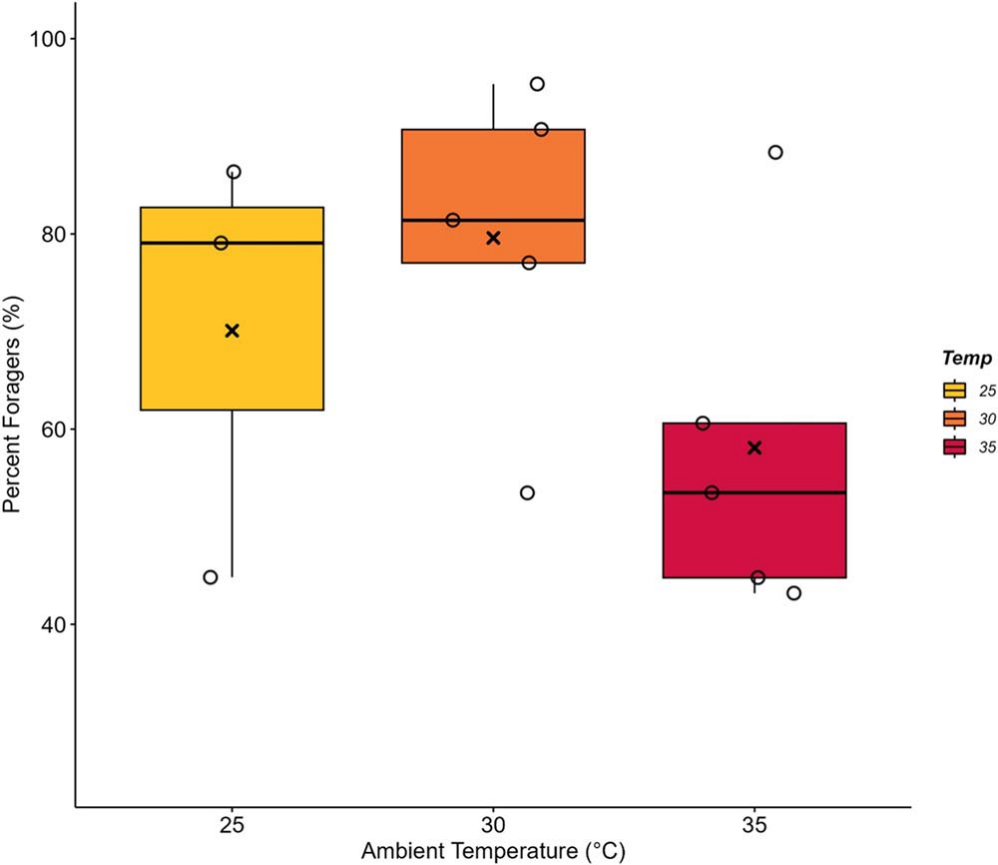
**The percentage of *B. impatiens* workers that forage shows no clear trend.** Bumblebee colonies of 43 workers were exposed to 25°C, 30°C or 35°C for 2-week periods (25°C, n = 3 colonies; 30, 35°C, n = 5 colonies). The percentage of tagged workers who engaged in foraging (10+ trips total) did not differ across T_a_ treatments (*P* = 0.230). Box plots represent percentiles with the black bar across equalling the median value. Also present are the individual data points to show the spread of the colonies tested. The average value is denoted by an (x) within each box.

On average, the percentage of tagged bees who participated in foraging was 70.09 ± 12.80% for colonies at 25°C, 79.59 ± 7.29% for colonies at 30°C and 58.08 ± 8.20% for colonies at 35°C with no significant effect of temperature between treatment groups (Fig. 1; F_2,10_ = 1.71, *P* = 0.230). Data for two of five colonies undergoing 25°C trials were unable to be recovered due to data saving failures of the Microsensys iID controller, lowering the sample size for that temperature group to n = 3. Power analysis revealed that larger sample size (16 colonies per group) would allow to detect differences in percent individuals engaging in foraging effort.

**Figure 2:**
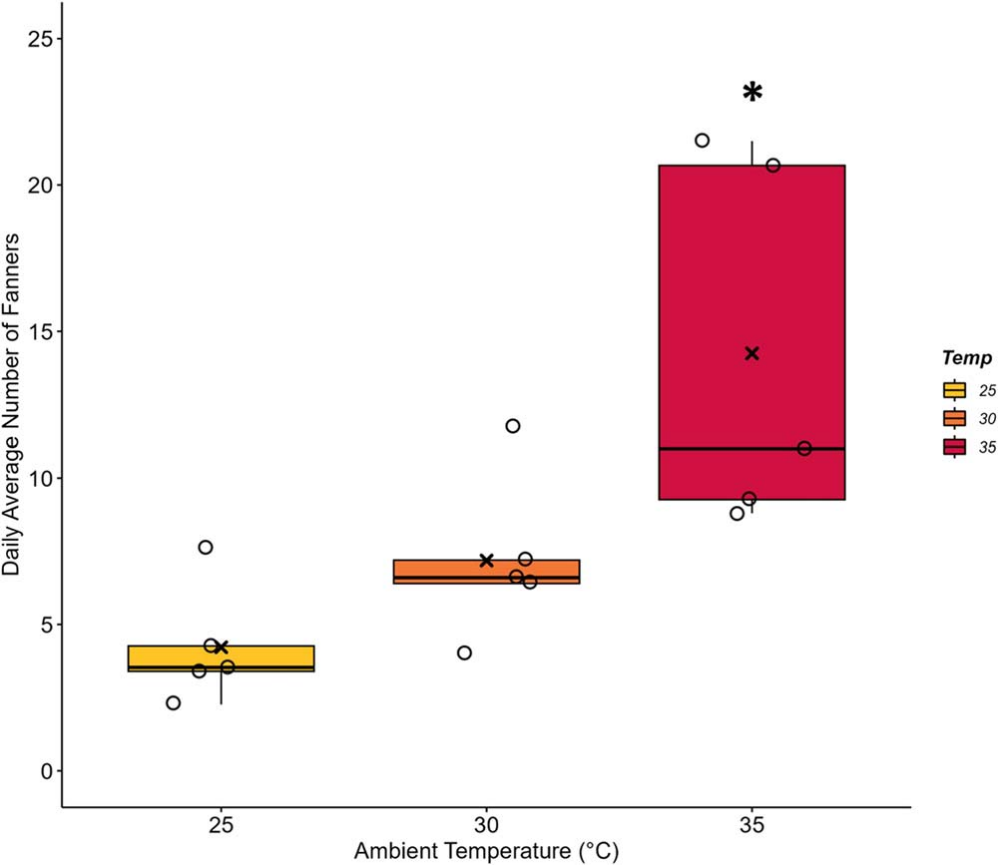
**Fanning incidence of *B. impatiens* workers increases under chronic heat stress.** Bumblebee colonies of 43 workers were exposed to 25°C, 30°C or 35°C for 2-week periods (n = 5 colonies per ambient temperature, T_a_). The incidence of fanning was quantified daily by counting workers engaged wing fanning for 10+ seconds within a 165-cm^2^ area overtop the brood. The daily totals were averaged across each T_a_ trial. Fanning incidence at 35°C was significantly higher than at lower T_a_ (*P ≤ 0.049). Box plots represent percentiles with the black bar across equalling the median value. Also present are the individual data points to show the spread of the colonies tested. The average value is denoted by an (x) within each box.

The average number of fanners was first tested using a mixed model accounting for colony as a random effect to determine if it changed over the 15 consecutive days of measurement (temperature, F_2,166_ = 7.94, *P* < 0.001; day, F_14,166_ = 0.49, *P* = 0.937; temperature*day: F_28,166_ = 1.92, *P* < 0.01). The significant interaction was due to the number of fanners that differed between Day 8 and Day 12 in the 25°C group only. We therefore further analyzed the mean number of fanners of all days combined and found it to be influenced by T_a_ (Fig. 2; F_2,12_ = 7.68, *P* = 0.007). The average number of fanners at 25°C (4.22 ± 0.91) was not different from the average at 30°C (7.19 ± 1.26; *P* = 0.516); however, the average number of fanners at 35°C (14.25 ± 2.82) was significantly higher than at both 25°C (*P* = 0.006) and at 30°C (*P* = 0.049). Power analysis indicated that only four colonies per group were sufficient to detect differences in fanning effort.

### Effect of ambient temperature on internal colony temperature

**Figure 3:**
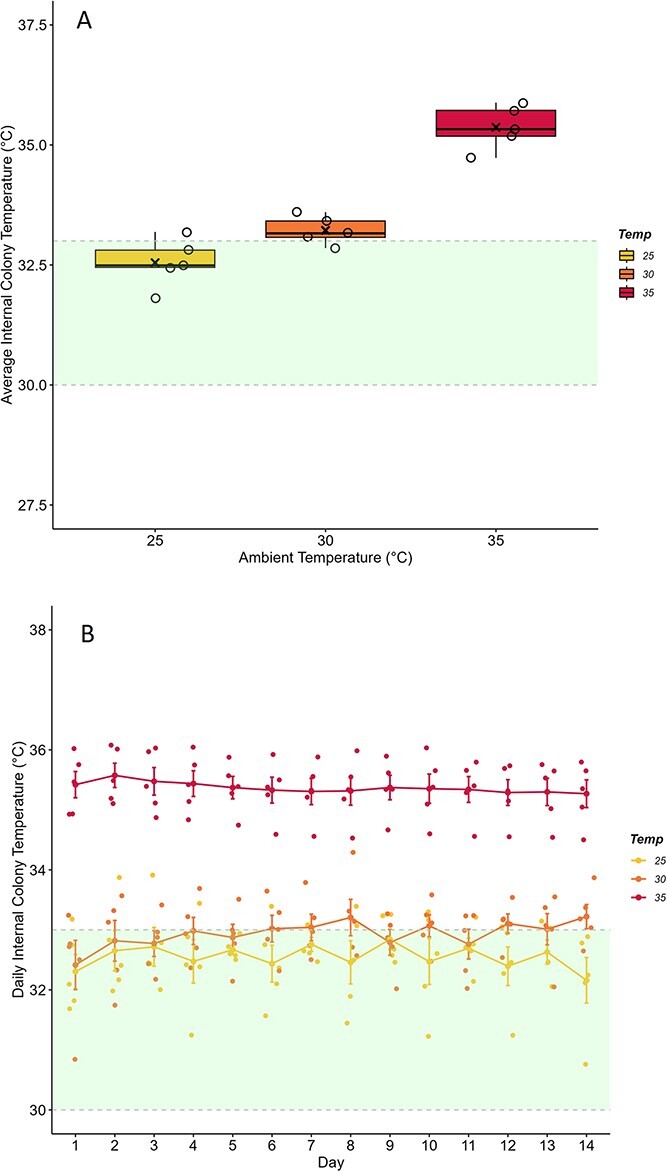
**Internal colony temperature cannot be maintained under chronic heat stress.** Bumblebee colonies (*B. impatiens*) of 43 workers were exposed to 25°C, 30°C or 35°C for 2-week periods (n = 5 colonies per ambient temperature, T_a_). Internal colony temperature was determined using iButtons placed within colony brood clumps. Optimal nest temperature (Barrow and Pickard, 1985; Vogt, 1986; Schultze-Motel, 1991; Heinrich, 2004) is represented by the shaded area on each panel. **A)** Average internal colony temperature, calculated across each T_a_ trial, was significantly higher at 35°C than at lower T_a_ (*P < 0.001). Box plots represent percentiles with the black bar across equalling the median value. Also present are the individual data points to show the spread of the colonies tested. The average value is denoted by an (x) within each box. **B)** The daily average internal colony temperature, calculated as the mean on each experimental day, showed an interact effect of T_a_ and day (*P* = 0.021). However, internal colony temperature did not differ on a daily basis within temperature groupings across 2-week expose periods with the exception of Day 1 having a lower value than Days 8 and 14 in the 30°C group (*P* < 0.05). Individual data points are scattered about the mean line and standard error points for each colony tested.

The internal colony temperature among the brood cells, as averaged across the 2-week experimental duration, was dependent on the T_a_ colonies were exposed to (F_2,12_ = 58.48, *P* < 0.001). The average T_n_ experienced by colonies at 25°C was 32.55 ± 0.23°C. Colonies at 25°C and at 30°C (average, 33.22 ± 0.13°C) had internal temperatures that were not significantly different from one another (*P* = 0.071). However, colonies who experienced 35°C had internal colony temperatures which hovered around ambient at 35.37 ± 0.20°C and were significantly higher than those at 25°C and 30°C (Fig. 3a; *P* < 0.001). For comparison, nest temperatures measured at the periphery away from the brood during initial trials were 28.81°C, 31.82°C and 35.62°C, for the 25°C, 30°C and 35°C treatment groups, respectively. Power analysis showed that small sample sizes (n = 2) was required to detect differences between groups.

Daily internal colony temperature remained fairly constant over the 2-week exposure period (Fig. 3B). A mixed model accounting for colony as random effect indicated a significant interaction between the temperature treatment and the day of measurement (temperature, F_2,156_ = 50.62, *P* < 0.001; day, F_13,156_ = 0.92, *P* = 0.534; temperature^*^day, F_26,156_ = 1.74, *P* = 0.021). The difference in nest temperature was observed for the 30°C group only where the temperature on Day 1 was slightly lower than Days 8 and 14 (*P* < 0.05).

### Effect of ambient temperature on adult emergence, mortality, abandonment and offspring production

**Figure 4:**
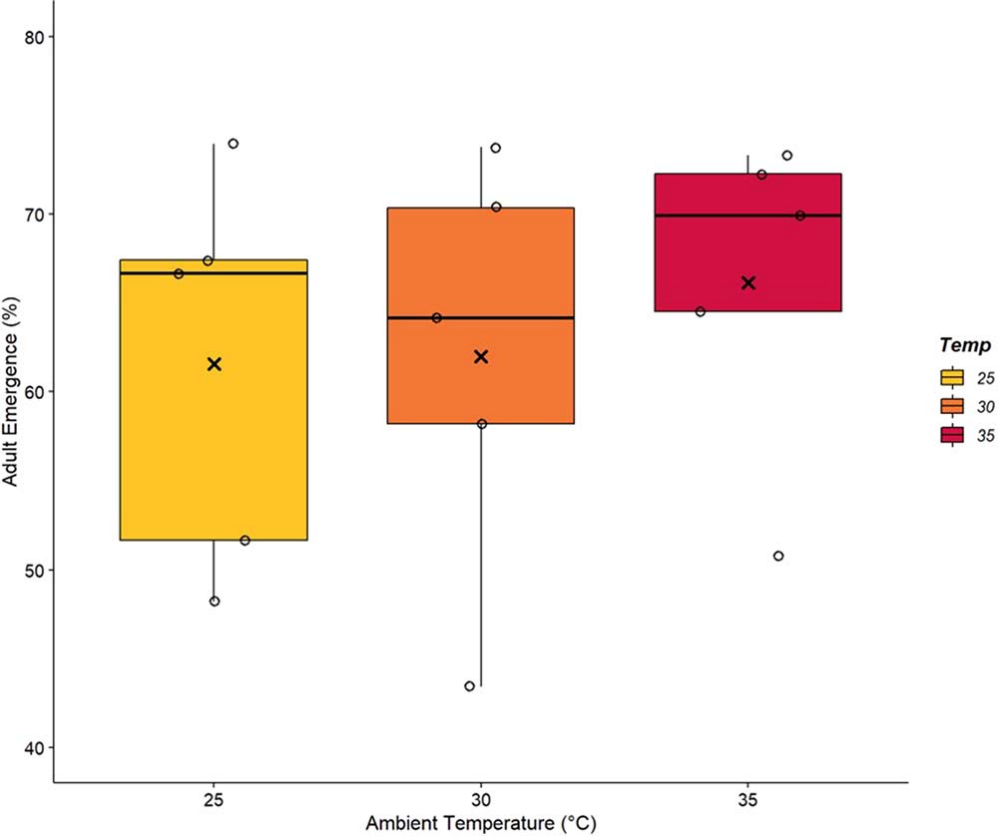
**Chronic heat stress does not alter the percent of adult emergence of *B. impatiens* colonies.** Bumblebee colonies of 43 workers were exposed to 25°C, 30°C or 35°C for 2-week periods (n = 5 colonies per ambient temperature, T_a_). The percentage of workers that emerged during an experimental trial was calculated to determine adult emergence, which ultimately did not depend on T_a_ (*P* = 0.765). Box plots represent percentiles with the black bar corresponding with the median value. Also present are the individual data points to show the spread of the colonies tested. The average value is denoted by an (x) within each box.

The number of workers who hatched over the course of an experimental trial, or percentage of adult emergence, was not dependent on T_a_ to which colonies were exposed (Fig. 4; F_2,12_ = 0.27, *P* = 0.765). On average, colonies at 25°C grew by 61.57 ± 4.95%, 30°C by 61.98 ± 5.35% and 35°C by 66.15 ± 4.14%. No male drones were found to have emerged in any of the trials. The detection of a significant effect between groups would have required very large sample sizes (n = 88) given the small differences observed between groups.

**Figure 5:**
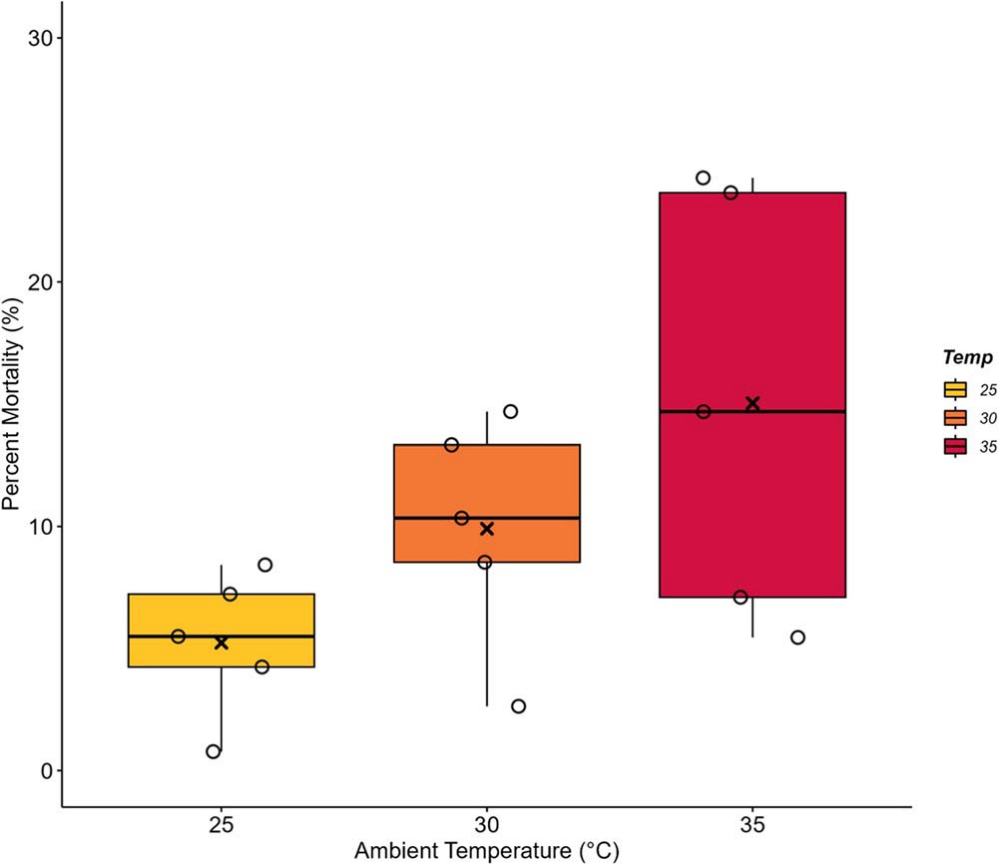
**Chronic heat stress does not change the percentage of mortality in *B. impatiens* colonies.** Bumblebee colonies of 43 workers were exposed to 25°C, 30°C or 35°C for 2-week periods (n = 5 colonies per ambient temperature, T_a_). The percentage of bees who were found deceased during or at the end of each experimental trial represents mortality rate. No significant differences were found between tested values of ambient temperature, T_a_ (*P* = 0.073). Box plots represent percentiles with the black bar across equalling the median value. Also present are the individual data points to show the spread of the colonies tested. The average value is denoted by an (x) within each box.

The percent mortality was not significantly influenced by T_a_, though resulting p-values indicate a potential trend (Fig. 5; F_2,12_ = 3.28, *P* = 0.073). Pairwise comparisons show that the average mortality at 35°C (15.03 ± 3.97%; *P* = 0.060) tended to be higher than at 25°C (5.23 ± 1.32%). At 30°C (9.91 ± 2.12%), however, there was no significant difference between the percent morality at either the 25°C or 35°C trials (*P* ≥ 0.402). It was also found that queens died before the end of the 2-week trial on four occasions; one from both the 25°C and 30°C experiments and two from the 35°C experiments. A power analysis indicated that larger sample sizes (n = 8) would be needed to detect a significant effect of temperature on percent mortality.

**Figure 6:**
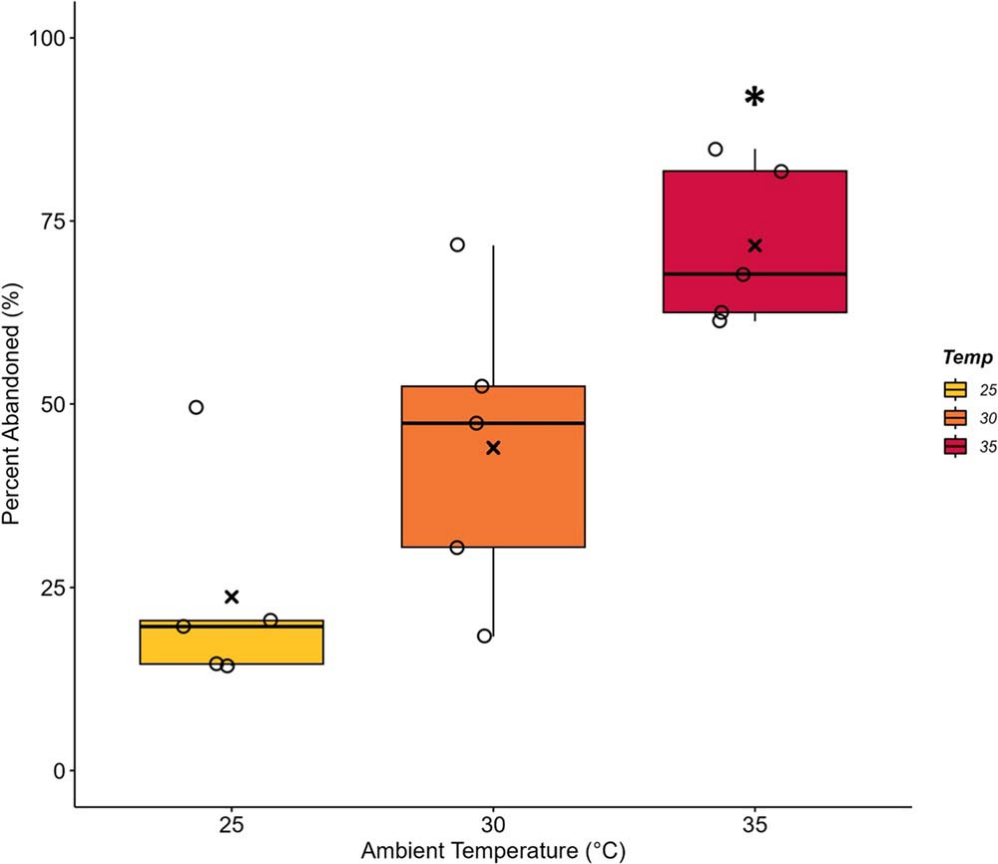
**Chronic heat stress results in a higher percentage of *B. impatiens* workers abandoning their colony.** Bumblebee colonies of 43 workers were exposed to 25°C, 30°C or 35°C for 2-week periods (n = 5 colonies per ambient temperature, T_a_). The percentage of workers who were found outside the colony and in the flight cage either deceased during or alive at the end of each experimental trial was used to quantify percent abandonment. This value was significantly higher at 35°C than at lower T_a_ (*P ≤ 0.044). Box plots represent percentiles with the black bar across equalling the median value. Also present are the individual data points to show the spread of the colonies tested. The average value is denoted by an (x) within each box.

The proportion of bees that abandoned the colony was dependent on the T_a_ experienced by the colony (Fig. 6; F_2,12_ = 11.4, *P* = 0.002). A greater percentage of bees were found to abandon the colony at 35°C (71.64 ± 4.92%) than at both 25°C (23.72 ± 6.60%; *P* = 0.001) and 30°C (44.05 ± 9.19%; *P* = 0.044), while this percentage did not differ between 25°C and 30°C (*P* = 0.150). Sample sizes of four colonies and more were sufficient to detect differences according to the power analysis.

**Figure 7:**
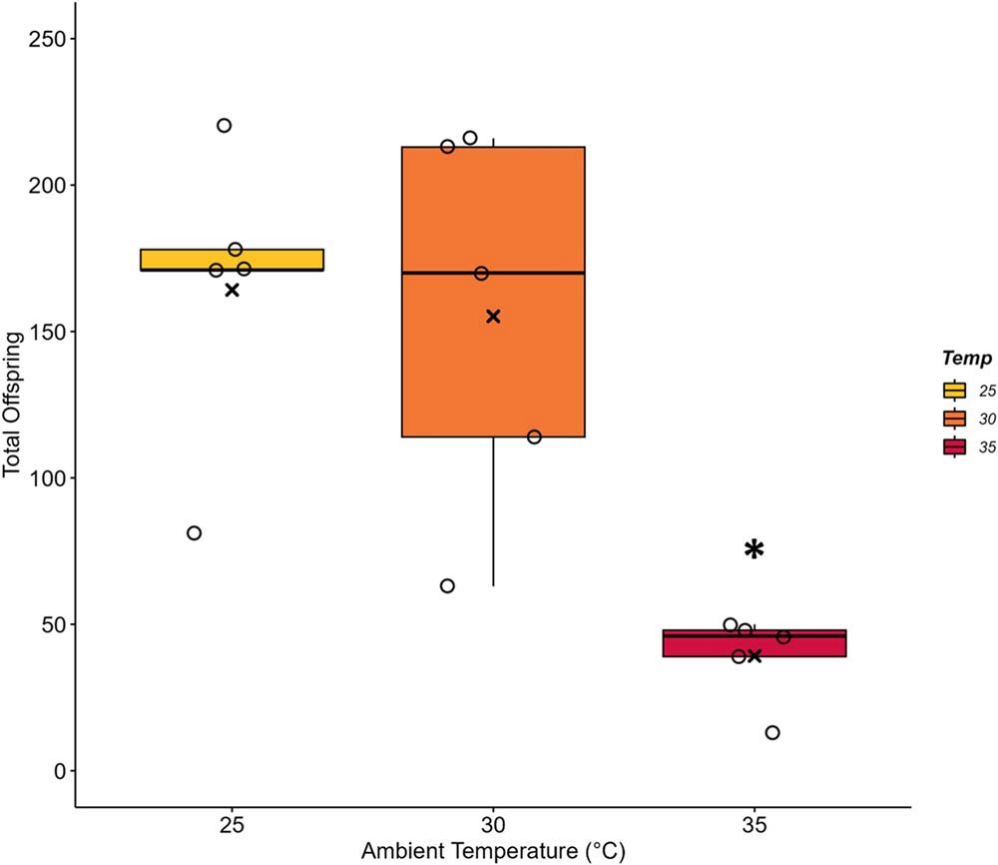
**Offspring production is negatively impacted by chronic heat stress.** Bumblebee colonies (*B. impatiens*) of 43 workers were exposed to 25°C, 30°C or 35°C for 2-week periods (n = 5 colonies per ambient temperature, T_a_). The total number of offspring present was dependent on T_a_ (*P* = 0.0026). The number of larvae and pupae present in dissected broods following the end of each experimental trial quantifies offspring production. Significantly fewer offspring were found at 35°C when compared to lower T_a_ (^*^P ≤ 0.007). Box plots represent percentiles with the black bar across equalling the median value. Also present are the individual data points to show the spread of the colonies tested. The average value is denoted by an (x) within each box.

The total number of larvae and pupae offspring produced within colonies varied with T_a_ (Fig. 7; F_2,12_ = 10.16, *P* = 0.003). On average, colonies at 25°C concluded the 2-week trial with 164.20 ± 22.71 unhatched offspring, the 30°C trials had 155.20 ± 29.54 and 35°C colonies ended with only 39.20 ± 6.81 total offspring. Total offspring counts did not differ between 25°C and 30°C trials (*P* = 0.955), but colonies experiencing 35°C had significantly lower numbers of offspring than both 25°C (*P* = 0.004) and 30°C (*P* = 0.007) trials. Power analysis indicated that only three colonies were required to detect differences among groups.

## Discussion

Extreme heat events and recent evidence of bumblebee species declines raise concern about the effects of chronic heat on the sustainability and success of bumblebee colonies. This study assesses how chronic exposure to high ambient temperature affects various aspects of a bumblebee colony's behaviour, growth and survival. To determine the capacity of bumblebee colonies to cope with chronic heat stress and whether it imposes compromises between foraging and fanning incidence and if adult emergence, mortality and nest temperature are affected, colonies underwent 2-week exposures to 25°C, 30°C and 35°C in a controlled environment. It was found that while foraging was not affected by high ambient temperature in our study, colonies under chronic thermal stress increased thermoregulatory fanning efforts but failed to reduce nest temperature. Furthermore, high temperatures resulted in increased colony abandonment as well as a reduction in the number of offspring produced under these conditions. Therefore, these results indicate that bumblebee colonies cannot cope with chronic high ambient temperature and that such conditions pose a significant risk to offspring production through reduced worker population and failed thermoregulation.

Bumblebee colonies experiencing chronic high temperature have greater proportions of workers engaging in the fanning behaviour, but it did not compromise the fraction of workers foraging in our study. The lack of effect of ambient temperature on foraging rates (measured as the proportion of tagged workers engaging in that behaviour) may be due, in part, to the limited number of individuals tagged (43 for each colony) and used to quantify this proportion. The power analysis conducted indicated that with the level of variation observed, where each treatment had one outlier colony, larger sample sizes would be required to reach strong conclusion. The possible decrease in foraging activity observed at 35°C should be scrutinized further given these limitations, and previous research supports that higher temperatures may reduce foraging activity in social bees (Kwon and Saeed, 2003; Couvillon *et al.,* 2010; Rami Reddy *et al.,* 2015), including undersimulated heatwaves (Hemberger *et al.,* 2023). Yet, other research demonstrates that rather than influencing the foraging rates of bumblebees, temperature will instead affect the type of resources that are collected, meaning that there are differing conditions favorable for either nectar or pollen foraging (Peat and Goulson, 2005). While foraging efforts were unaffected by ambient temperature, fanning effort increased under chronic exposure to 35°C. Short-term observations of bumblebee colonies under heat stress demonstrate that fanning efforts increase when ambient temperature exceeds 30°C (Vogt, 1986; Wynants *et al.,* 2021). For *B. impatiens* colonies, chronic exposure to high ambient temperature also leads to long-term fanning effort, which was observed to be sustained across the full 2-week exposure period. Given that 35°C exceeds the range of optimal nest temperature determined in previous studies (Barrow and Pickard, 1985; Vogt, 1986; Schultze-Motel, 1991; Heinrich, 2004), thermoregulatory fanning under these conditions is deployed as a method to reduce nest temperature in attempt to prevent detrimental effects in unhatched offspring. In honeybees, larval contact plays a role in increasing the probability of worker fanning behaviour (Cook *et al.,* 2016) and individual worker bumblebees possess varying thermal thresholds as a behavioural trigger, providing a graded colony response to combat increases in temperature and maintain thermal homeostasis (Weidenmüller *et al.,* 2002; Weidenmüller, 2004).

Despite the high incidence of fanning exhibited by our tested colonies at 35°C, their efforts were unsuccessful. Internal colony temperatures rose to ambient temperature within the first day of the 2-week experiments and colonies were unable to mitigate this thermal threat. The laboratory conditions used where no insulation was present inside the nest could have prevented them from adequate thermoregulation, but shorter-term exposure of colonies with and without insulation also show that nest reached environmental temperature at 35°C (Bretzlaff *et al.,* 2023). At 25°C and 30°C, internal colony temperature remained within the known optimal nest temperature range (Barrow and Pickard, 1985; Vogt, 1986; Schultze-Motel, 1991; Heinrich, 2004) over the 2-week period, further demonstrating that bumblebee colonies struggle to thermoregulate their nests under both chronic high ambient temperature conditions and acute thermal stress; the latter also resulting in increased energy expenditure (Vogt, 1986; Bretzlaff *et al.,* 2023). Fanning, which employs wing muscles within the thorax, inevitably produces heat as a result of mechanical work (Heinrich and Kammer, 1973). Thorax temperature of workers in a colony is known to increase in response to cold exposure (Macías-Macías *et al.,* 2011) and during incubation (Heinrich, 1972b) as heat is transferred to warm the brood. Over a temperature range of 20°C to 34°C, however, the abdominal temperatures of incubating *B. terrestris* queens and workers are not correlated to T_a_ (Wynants *et al.,* 2021). Overall, long-term fanning efforts employed by colonies are unsuccessful at regulating T_n_ under chronic heat stress.

Warm conditions, where fanning behaviour provides little benefit to thermoregulation, put colony growth at risk by affecting offspring production and survival. Heat stress has been found to decrease nest mass growth and slow brood production, especially in bumblebee colonies experiencing nutritional stress as well (Vanderplanck *et al.,* 2019). We found that the number of bees which emerged during a 2-week exposure period was relatively unchanged by ambient temperature. Given that each colony tested began an experimental trial with similar brood sizes, our findings for adult emergence suggest that the eclosion of existing pupae is not significantly impacted by chronic high ambient temperature. Mean adult emergence was similar across temperatures and the level of variation observed would require very large sample size (n = 88) to detect differences. Instead, prolonged exposure to high ambient temperature may have greater potential to increase the mortality rates of adult colony members as indicated by an apparent increase between 25°C and 35°C which approached significance. In this case, the modest sample size used limits our conclusion as the power analysis indicates that eight colonies would be required. Furthermore, the number of unhatched larvae and pupae collected after a 2-week exposure period was dramatically reduced at 35°C, implying that chronic heat stress imposes negative effects on either egg laying or the early instar development of new offspring. Previous works further emphasize the direct effects of chronic exposure to high T_a_ during rearing on bee survival and development, such as lowering the colony's investment in offspring production in bumblebees (Vanderplanck *et al.,* 2019), reducing or preventing pupal emergence in honeybees (Groh *et al.,* 2004), decreasing adult longevity in honeybees and Megachilidae bees (CaraDonna *et al.,* 2018) as well as causing morphological deformities in honeybees (Groh *et al.,* 2004; Medina *et al.,* 2018). Previous work on honeybees also shows the importance of relative humidity for egg hatching and that low relative humidity within the nest can result in reduced worker survival (see Abou-Shaara *et al.,* 2017). We did not monitor the microenvironment of the colony at the brood level other than temperature, and further work would benefit from monitoring such a variable. Additionally, the interaction between environmental temperature and relative humidity level could further impact colony success, although for species, such as honeybees, they appear quite resilient other than in the driest and warmest climates (Abou-Shaara *et al.,* 2017), and the impact of humidity level on bumblebee foraging is thought to be mostly due to flower nectar production (Peat and Goulson, 2005). Finally, high temperature also leads to increased abandonment of the colony by workers. One limitation in our measurement of abandonment is that bees which died foraging within the flight cage were also considered, but the proportions reported are dominated by bees that truly abandoned the nest and that were found alive in the flight cage at the end of the experiment. The overall significant increase in the percentage of workers who were found outside of the colony at 35°C suggests that chronic heat stress drives individuals to leave the colony, but the permanence of such a phenomenon remains in question once the heat stress is removed. Abandonment may thus further impact colony success by reducing the number of individuals available to perform behaviours that are essential to colony function such as foraging, thermoregulation and nest maintenance. Impairments to colony function, as a result a sublethal environmental stressors, are linked with reduced colony success (Bryden *et al.,* 2013), therefore, combined increases in worker abandonment and reduced offspring production may act to have the greatest impact on bumblebee colony success under chronic heat stress.

The results obtained from our laboratory study inform about the capacity of bumblebee colonies to cope with chronic warm temperatures, but there are several distinctions when transposed to natural settings. Conditions used correspond more to surface or aboveground nesting that provide minor buffering from the environment. Underground nest sites are the most frequently observed nesting strategies across multiple bumblebee species, including *B. impatiens* (Colla *et al.,* 2014). However, surface or aboveground nest sites combined are almost as frequently reported for natural settings and even more frequent when nesting in artificial nest such as human made structures (Liczner and Colla, 2019). Aboveground temperatures can cause wide fluctuations in nest temperature as indicated by experiments on empty nest boxes where temperatures inside can be seen to vary by as much as 24°C throughout the day (Mullan, 2022). Long-term monitoring of bumblebee colonies from various species in aboveground nest boxes show that some species will have brood temperature experiencing large diurnal fluctuations while others appear to regulate it much more tightly (Gradišek *et al.,* 2023). It is also noteworthy that brood temperature reported from this study rose above 35°C. Thus, bumblebee colonies can experience nest environments that exposes them to thermal challenges akin to our experiments, but may buffer their exposure through the insulation present or possibly added, plus they usually experience relief during the diurnal cycles, which likely affect the severity and the thermal challenge. Nevertheless, our study provides the realm of potential response and an indication of the hierarchy of traits affected by chronic high temperature exposure.

## Conclusions

Increasing global temperature and more frequent occurrences of heat wave events are well documented to have negative broad-scale impacts on bumblebee species’ ranges and richness (Kerr *et al.,* 2015; Soroye *et al.,* 2020), as well as abundance (Rasmont and Iserbyt, 2012), respectively. The underlying causes of such impacts, however, remain poorly understood, especially when considering the effects of prolonged heat exposure on colony success. Our study investigating the effects of constant high temperature exposure on bumblebee colonies shows both direct and indirect effects to colony success. Temperatures which exceed optimal thermal nest conditions result in elevated nest temperature that cannot be mitigated by the increased fanning efforts observed. Increases in heat-related mortality in existing workers and unhatched larvae and pupae may either be a direct effect of exposure to high ambient temperature or a combination of indirect consequences of prolonged exposure. While the number of foragers did not significantly decline at high ambient temperature in the timeframe of the experiment, the greater allocation of workers engaging in fanning at 35°C, combined with greater mortality and fewer offspring produced, may ultimately leave fewer individuals to carry out tasks such as fanning and nest maintenance, which are energetically expensive (Vogt, 1986). Nutrient-deficient colonies of bumblebees both produce fewer individuals (Vaudo *et al.,* 2018; Vanderplanck *et al.,* 2019) and may cause conflicts between thermoregulatory and foraging task allocation (Stewart *et al.,* 2021). If unable to provide the necessary resources through foraging, and if coupled with increased abandonment of workers, heat stress events may ultimately result in fewer individuals to care for the current brood. The hindered success at high ambient temperature may be explained by a combination of the above direct and indirect effects of thermal stress as possible explanation for observed declines to date. Our study presents the impact of constant chronic heat stress on whole bumblebee colonies, and the timeline and extent of these effects for colonies exposed to conditions mirroring natural fluctuations in colony microclimate are further needed.

## Supplementary Material

Web_Material_coae006

## Data Availability

The data underlying this article are available in the article and in its online supplementary material.
